# Diagnostic Accuracy of Single-Lead Electrocardiograms Using the Kardia Mobile App and the Apple Watch 4: Validation Study

**DOI:** 10.2196/50701

**Published:** 2023-11-23

**Authors:** Kristina Klier, Lucas Koch, Lisa Graf, Timo Schinköthe, Annette Schmidt

**Affiliations:** 1 Institute of Sport Science University of the Bundeswehr Munich Neubiberg Germany; 2 CANKADO GmbH Ottobrunn Germany; 3 Research Center for Smart Digital Health University of the Bundeswehr Munich Neubiberg Germany

**Keywords:** accuracy, electrocardiography, eHealth, mHealth, mobile health, app, applications, mobile monitoring, electrocardiogram, ECG, telemedicine, diagnostic, diagnosis, monitoring, heart, cardiology, mobile phone

## Abstract

**Background:**

To date, the 12-lead electrocardiogram (ECG) is the gold standard for cardiological diagnosis in clinical settings. With the advancements in technology, a growing number of smartphone apps and gadgets for recording, visualizing, and evaluating physical performance as well as health data is available. Although this new smart technology is innovative and time- and cost-efficient, less is known about its diagnostic accuracy and reliability.

**Objective:**

This study aimed to examine the agreement between the mobile single-lead ECG measurements of the Kardia Mobile App and the Apple Watch 4 compared to the 12-lead gold standard ECG in healthy adults under laboratory conditions. Furthermore, it assessed whether the measurement error of the devices increases with an increasing heart rate.

**Methods:**

This study was designed as a prospective quasi-experimental 1-sample measurement, in which no randomization of the sampling was carried out. In total, ECGs at rest from 81 participants (average age 24.89, SD 8.58 years; n=58, 72% male) were recorded and statistically analyzed. Bland-Altman plots were created to graphically illustrate measurement differences. To analyze the agreement between the single-lead ECGs and the 12-lead ECG, Pearson correlation coefficient (*r*) and Lin concordance correlation coefficient (*CCC_Lin_*) were calculated.

**Results:**

The results showed a higher agreement for the Apple Watch (mean deviation QT: 6.85%; QT interval corrected for heart rate using Fridericia formula [QTcF]: 7.43%) than Kardia Mobile (mean deviation QT: 9.53%; QTcF: 9.78%) even if both tend to underestimate QT and QTcF intervals. For Kardia Mobile, the QT and QTcF intervals correlated significantly with the gold standard (*r_QT_*=0.857 and *r_QTcF_*=0.727; P<.001). *CCC_Lin_* corresponded to an almost complete heuristic agreement for the QT interval (0.835), whereas the QTcF interval was in the range of strong agreement (0.682). Further, for the Apple Watch, Pearson correlations were highly significant and in the range of a large effect (*r_QT_*=0.793 and *r_QTcF_*=0.649; P<.001). *CCC_Lin_* corresponded to a strong heuristic agreement for both the QT (0.779) and QTcF (0.615) intervals. A small negative correlation between the measurement error and increasing heart rate could be found of each the devices and the reference.

**Conclusions:**

Smart technology seems to be a promising and reliable approach for nonclinical health monitoring. Further research is needed to broaden the evidence regarding its validity and usability in different target groups.

## Introduction

Digitalization and technological progress are extending to more and more areas of life, including the fitness and health care sectors. The number of digital apps for smartphones, fitness trackers, or smartwatches that allow users to assess and evaluate individualized fitness, health, and lifestyle data is constantly increasing [1]. With the release of the Apple Watch 4 as one of the first smartwatches to include electrocardiogram (ECG) function in 2018, smartphone-based systems that enable users to record single-lead ECGs on their own have become very popular [2]. Such devices are designed to help prevent cardiovascular diseases, for example, by identifying cardiac arrhythmia at an early stage and thereby preventing a stroke.

According to the German Stroke Foundation [3], around 270,000 people endure a stroke in Germany every year. This is why strokes and their health consequences are the third most common cause of death in Germany and even one of the most common causes of death worldwide [4]. In the age group of over 60 years, the quota of those affected amounts to almost 80% [3]. One of the reasons for this high mortality rate is, among other things, that cardiac arrhythmias such as atrial fibrillation are detected too late. This in turn might be due to the fact that atrial fibrillation can be asymptomatic and, therefore, is often unnoticed. In addition, it occurs only intermittently in many cases, which is also why it is difficult to detect. However, atrial fibrillation can increase the risk of enduring a stroke by up to 5 times [5]. In total, 15% to 20% of all strokes are due to this type of cardiac arrhythmia. This means that a stroke due to atrial fibrillation happens almost every 10 seconds [5]. Any cardiac arrhythmia can be detected by ECG diagnostics, and consequently, suitable and timely treatment by a doctor can prevent a stroke.

As stated, affordable compact devices for recording a single-lead ECG at home entered the market recently [6]. Unlike the conventional ECG, a diagnosis is no longer dependent on the symptoms to occur at the time of measurement in a doctor’s office. Rather, a patient may notice symptoms such as tachycardia or shortness of breath and is able to carry out an ECG measurement immediately him- or herself.

Especially in sports science, the ECG is also one of the most important diagnostic tools in terms of the determination of the individual physical performance, the reproduction of loads, or the exclusion of contraindications referring to the cardiovascular system [7]. Hereby, it is important to distinguish athletes’ usual training-related changes from unusual and nontraining-related potentially pathological abnormalities. For example, in endurance athletes, ECG changes in the form of sinus arrhythmias and sinus bradycardia are most common. Further, changes in the ventricular complexes or during repolarization, as well as earlier repolarization, can occur [8].

The 12-lead ECG is the current reference method (“gold standard”) for recording cardiovascular parameters. Although the 12-lead ECG is used in clinical settings, its complex structure presents certain economic and practical limitations that need to be considered [9,10]. From a sports cardiological perspective, single-lead ECGs have the potential to be an alternative to the established 12-lead ECG. The improvement of wearable devices’ measured value density and quality is the main reason for this assessment [11,12]. Comparably, compact single-lead ECGs for smartphones and smartwatches are much easier and more time-efficient to use by assessing all relevant heart (rate) parameters [13]. Although the manufacturers claim that this smart technology can be used in both clinical and nonclinical settings, less is known about the measurements’ validity and reliability from a scientific perspective [14,15]. Therefore, we aimed to examine the measurements’ accuracy of the Kardia Mobile App and the Apple Watch 4 in comparison to a 12-lead gold standard ECG. Aside, we analyzed whether the devices’ measurement error correlates with an increasing heart rate.

## Methods

### Participants

Data collection took place in our laboratory. Participants were recruited via advertisement. In order to participate, individuals must be at least 18 years old and in good physical health. Persons with cardiovascular diseases were excluded from this study. In total, 100 adults took part, with 81 complete measurements that could be incorporated in the statistical analysis. The age of the sample ranged between 19 and 78 (mean 24.89, SD 8.58) years; of the 81 participants, 23 (28%) were female and 58 (72%) males.

### Study Procedure

This study was designed as a prospective quasi-experimental 1-sample measurement, in which no randomization of the sampling was carried out. Each data collection began with the gluing of disposable electrodes to the chest and limbs and the cabling of the 12-lead ECG. If the signal was free of interference, a 10-second measurement was started in the lying position. After this successful reference measurement, the following measurement via the Kardia Mobile was carried out. Finally, the last measurement via the Apple Watch was conducted. This study’s procedure respective measurement order as well as the lying position were the same for all subjects. To address the possibility of bias due to a consistent test order, several measurements were conducted as a pretest for the final study procedure. The design of this study is based on the recommendations for implementing validation studies of diagnostic devices [16,17].

### Ethical Considerations

This study was conducted according to the guidelines of the Declaration of Helsinki and approved by the ethics committee of the University of the Bundeswehr Munich, Germany (June 4, 2018). Informed consent was obtained from all subjects participating in this study. This included comprehensive information about the course of this study, data storage and use, and possible health risks during or after the examination. Participants consented to the collection, storage, and analysis of their personal data. Each participant was given the opportunity to withdraw from this study at any time and for any reason. This includes the complete deletion of all data already collected from the participant and copies thereof unless they have already been anonymized. No compensation of any kind was provided for participation in this study.

### Materials

#### Custo Cardio 300

**Figure 1:**
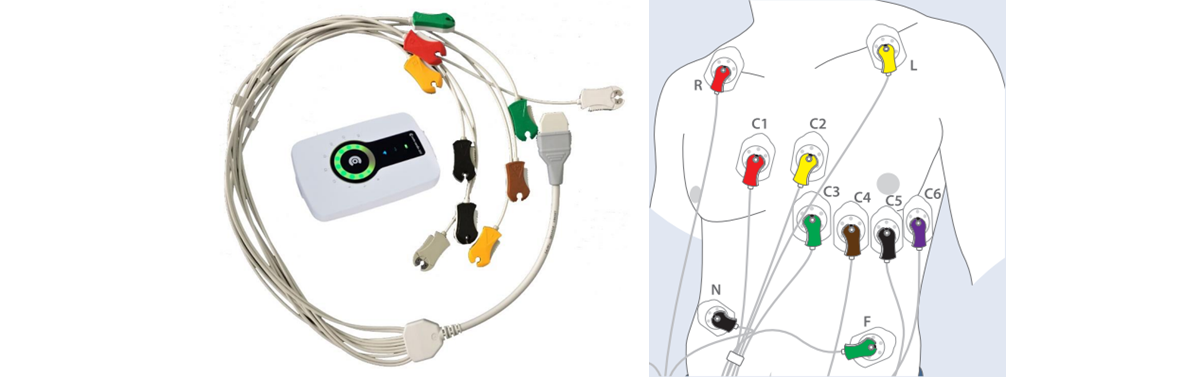
12-lead ECG device Custo Cardio 300 (left) and placement of its disposable electrodes (right). ECG: electrocardiogram.

We used the Custo Cardio 300, a valid 12-lead ECG, as the reference device in our study (Figure 1) [18]. It is manufactured for medical centers and hospitals by the German company custo med GmbH and is linked to their foreign analysis software *custo diagnostic*.

The device records the individual’s ECG via 12 leads comprising of 6 leads of the limbs (I, II, III, augmented vector left, augmented vector right, and augmented vector foot) and 6 leads of the chest wall (C_1_, C_2_, C_3_, C_4_, C_5_, and C_6_) [19]. Referring to Goldberger [20], the disposable electrodes for the limb leads were placed at the wrists and ankles. According to Wilson [21], the chest wall leads were placed as follows: C_1_ in the fourth intercostal panel at the right side of the sternum, C_2_ in the fourth intercostal panel at the left side of the sternum, C_3_ between C_2_ and C_4_ on the fifth rib, C_4_ in the fifth intercostal panel at the intersection with the left midclavicular line, C_5_ at the same level as C_4_ on the anterior axillary line, and C_6_ at the same level as C_4_ on the midaxillary line. The electrodes are connected to the ECG device by a 10-wire patient cable with colored clips. The integrated LED ring of the device provides visual information about the signal quality of each individual lead. If electrodes are not applied to the subject, the relevant LEDs light up red. If every lead is correctly applied, the corresponding LEDs light up green. The recording of the ECG can be started by either using the button on the device or setting it in manually via the software on the computer. Data can be transferred via Bluetooth, wireless local-area network, or USB port. When the 10-second-resting-ECG mode is completed, the recording is automatically ended, saved, aligned, and displayed in the software.

#### Kardia Mobile and Kardia App

**Figure 2:**
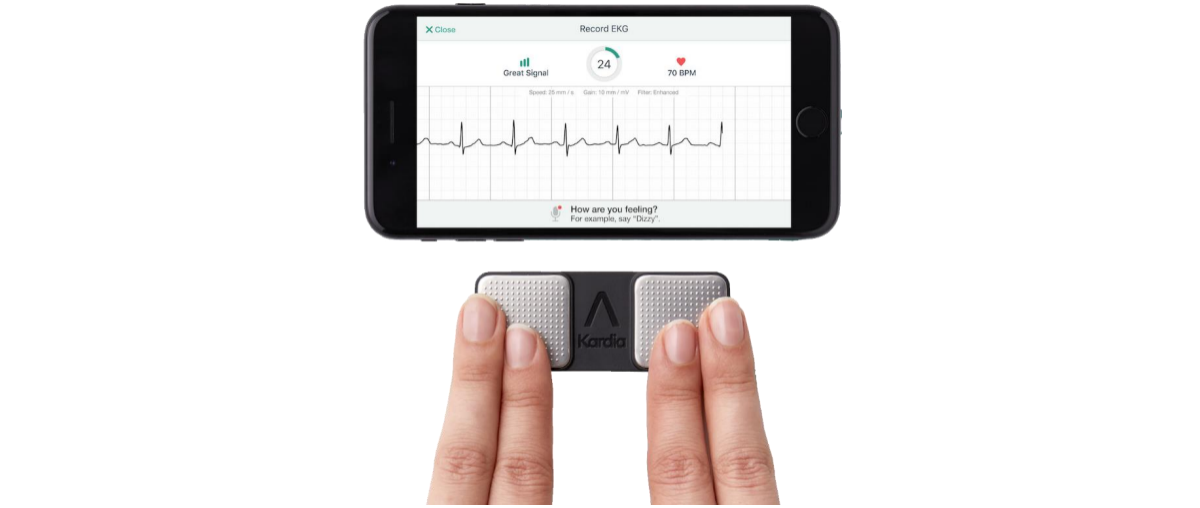
Recording of an ECG with the Kardia Mobile device connected to the Kardia App. ECG: electrocardiogram.

The second device we used was the single-lead ECG Kardia Mobile combined with the associated app for any smartphone or tablet (Figure 2) [22]. Its manufacturer AliveCor is a medical device and artificial intelligence company that sells ECG hardware and software for mobile devices. The company is the first company that has US Food and Drug Administration (FDA) approval for a medical device accessory for the Apple Watch and pioneered the development of FDA-approved machine learning techniques. The Kardia Mobile was approved by the FDA in December 2012 and is Conformité Européene marked [13]. According to AliveCor, it is one of the most clinically validated mobile compact ECG on the market. The Kardia Mobile together with the Kardia app can record, display, and transmit a single-lead ECG. Its algorithm has been approved by the FDA solely for the analysis to detect atrial fibrillation and a normal sinus rhythm, although it also claims to indicate whether bradycardia or tachycardia is present. The device measures 8.2 cm (length)  3.2 cm (width)  0.35 cm (depth) with a weight of 18g (including a 3.0V, CR2016 battery). The 2 square stainless-steel electrodes comprise an area of 9cm^2^. Via the supplied mounting plate, it can directly be attached to the back of the smartphone. With normal use, the device has an operating time of about 200 hours or 12 months and an estimated shelf life of 2 years.

To record an ECG, a smartphone or tablet running the Kardia app is needed. For the measurement, first, the “Record ECG” option in the app needs to be selected. Then, the index and middle fingers are placed on the electrodes of the device with the right hand on 1 electrode and the left hand on the other electrode. As soon as there is good contact with the electrodes, the app starts recording automatically*.* Kardia Mobile transmits the data wirelessly via ultrasound to the smartphone, displaying real-time heart rate as well as an ECG waveform similar to lead I of a 12-lead ECG. According to the manufacturer, the device should be at a distance of up to 30 cm from the smartphone or tablet. Normally, the recording lasts 30 seconds. Immediately after completion, an evaluation is available stating whether the ECG is within the normal range, it cannot be classified, atrial fibrillation is detected, or if the recording is unreadable. The app classifies the ECG as normal if the heart rate is between 50 and 100 beats per minute (bpm); none or only a few abnormal beats are present; and if form, timing, and duration correspond to a sinus rhythm. Finally, the Kardia app allows us to export the ECG as a PDF file in order to save and send it via email to the user oneself or his or her health care provider.

#### Apple Watch 4

**Figure 3:**
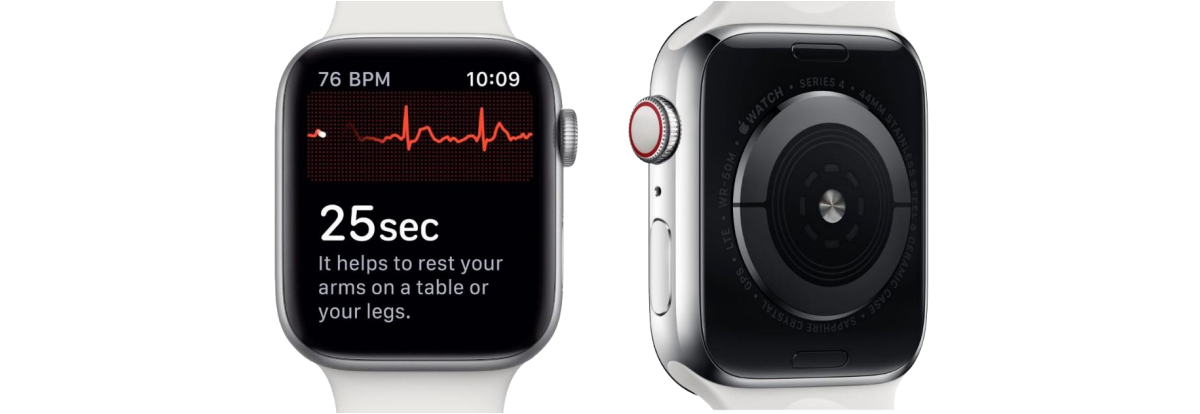
Single-lead ECG Apple Watch 4. BPM: beats per minute; ECG: electrocardiogram.

As third, we used the *Apple Watch Series 4*, which is, without further gadgets, also able to record a single-lead ECG (Figure 3) [23].

For the ECG recording, an Apple Watch 4 or later running at least watchOS 5.2 as well as an iPhone running at least iOS 12.2 are needed. The device is the standard model and measures 4.0 cm (length)  3.4 cm (width)  1.07 cm (depth) with a weight of 30.01 g. It has a battery life of up to 18 hours. The required electrodes for the ECG are integrated with the so-called “Digital Crown,” for example, the small rotary knob on the side and on the back of the watch. By touching the Digital Crown with the index finger of the other hand, the circuit between the heart and the arms is closed so that the electrical impulses of the heart can be measured, comparable to lead I according to Einthoven [23]. The Apple Watch’s ECG provides information on heart rate and heart rhythm and, thus, enables the classification of sinus rhythm and atrial fibrillation. Unlike the automatic heart rhythm monitoring several times a day via the optical pulse sensor, the ECG function must be actively started. Meanwhile, the 30 seconds of recording, a typical ECG curve, a countdown, and the heart rate are displayed on the watch. Afterward, a classification is apparent, and the data are directly transmitted to the app on the smartphone. In line with the Kardia Mobile, the ECG is classified as normal if the heart rate is between 50 and 100 bpm and a stable sinus rhythm could be detected. The results of the ECG recording can be viewed via the health app on the smartphone or exported as a PDF file.

### Data Analysis

#### Data Export

**Figure 4:**
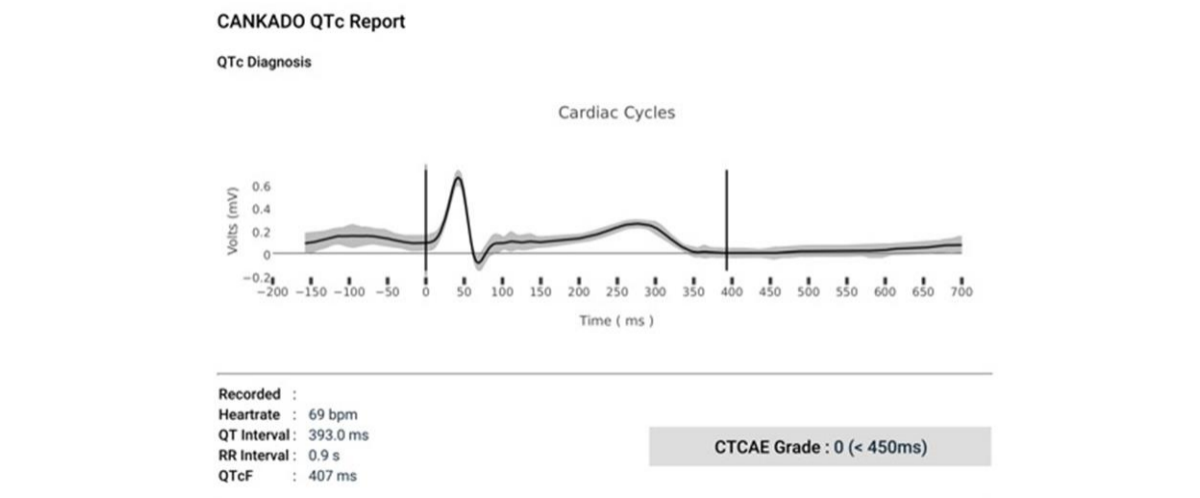
ECG Analysis via QTc Tracker. The algorithm of the QTc Tracker is based on Fridericia formula, which calculates the quotient from the QT interval and the cube root of the RR interval (QT/(RR) 1/3). CTCAE: Common Terminology Criteria for Adverse Events; ECG: electrocardiogram; QTc: QT interval corrected for heart rate.

We exported the recordings of the 12-lead ECG via the custo diagnostic software. As this PDF export already provides all relevant parameters such as QT, QT interval corrected for heart rate using Fridericia formula (QTcF) intervals, and heart rate, no further data processing was required. The QT interval comprises the time from the start of the Q wave to the end of the T wave. Based on Fridericia formula [24], the heart rate corrected QT interval (QTc) can be determined [25]. The data export of the compact single-lead ECGs was similar for both devices. Besides the heart rate and the classification of the ECGs, the created PDFs also comprised the 30-second recording only as a graph with a waveform comparable to a lead I ECG. Therefore, it was necessary to calculate the QT and QTcF intervals from these graphical ECG waves with the help of appropriate software. Hereby, we used the Beta version of the app *QTc Tracker* by CANKADO GmbH, which is specially designed to extract data from single-lead ECGs. The app’s algorithm is the winning algorithm of the “2017 PhysioNet/CinC Challenge” [26]. The app matches all data points of 1 measure and averages them as an ECG curve while deviating data points are presented as gray area around the averaged curve (Figure 4) [27]. Furthermore, the program suggests the beginning and the end of the QT interval, which can be manually corrected if needed. If both markers have been set, it finally calculates the RR, the QT, and the QTcF intervals. The QTc Tracker’s output function is limited to the parameters mentioned, preventing the evaluation of other ECG parameters such as the P wave or ST segment. A first description and examination of the QTc Tracker in the oncological routine has recently been published [28].

#### Statistical Analysis

Statistical Analysis was carried out using the data processing programs Microsoft Excel and SPSS (version 27; IBM Corp). After the descriptive analysis, we first graphically analyzed the agreement between the single-lead and the reference ECG via Bland-Altman plots [29,30]. The x-axis is the mean of both devices, and the y-axis represents the 12-lead minus the single-lead ECG with the line of equality (LoE) plotted at zero. The dotted lines (LoA [limit of agreement]) are 1.96 SDs from the mean, and the thin lines are the 95% CI of the mean. Second, to examine the correlation between the devices, we calculated Pearson correlations and interpreted the results according to Cohen [31]. The level of significance was set a priori at α<.05. However, the Pearson correlation coefficient does not consider the location shift (parallel shift of the degrees of regression compared to the bisecting line) nor the scale shift (rotation of the regression line so that it has a different slope than the bisecting line), and further allows no conclusion about the intraindividual concordance (agreement of measured values of the same person) [32]. Therefore, third, we calculated Lin concordance correlation coefficient (*CCC_Lin_*) as it includes both aspects. *CCC_Lin_* weights the correlation coefficient *r* according to Pearson with a correction term that, including mean and SD, corresponds to the deviation from the bisecting line (see equation 1 below) [33]. The results were classified in addition to Cohen κ [34]. If the ECG devices were completely in agreement, both the location and scale shift (accuracy) would be 0, and the precision (correlation) *r*=1, that is, *CCC_Lin_*=1. Mathematical formula for *CCC_Lin_* is as follows:




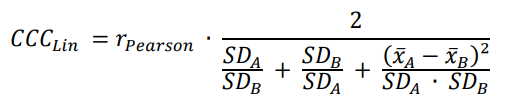


**(1)**

## Results

### Descriptive Analysis

We included the heart rate and QT and QTcF intervals in our analysis. Referring to the 12-lead ECG, the participants heart rate ranged between 38 and 98 (mean 66.44, SD 11.52) bpm. Descriptive statistics of QT and QTcF are presented in Table 1. The mean QT interval of the 12-lead reference device was 387.89 (SD 27.1) ms. On average, the Kardia Mobile deviated 37.27 ms from the reference, which was up to 9.53% (SD 3.62%) mean deviation. The Apple Watch differed on average by 25.89 ms, which was with 6.85% (SD 4.02%), a smaller mean deviation. Regarding QTcF, the reference device measured 399.09 (SD 18.2) ms while both single-lead ECGs assessed shorter interval durations. The Kardia Mobile differed on average by 39.04 ms, which means a 9.78% (SD 3.76%) deviation. The Apple Watch differed on average by 29.52 ms, which means with 7.43% (SD 4.17%), a smaller deviation from the reference device. Taken together, the single-lead ECGs tend to underestimate both the QT as well as the QTcF intervals compared to the 12-lead gold standard.

**Table 1:** Descriptive statistics of QT and QTcF^a^ intervals. Quasi-experimental 1-sample measurement with no randomization (81 healthy adults; mean age 24.89, SD 8.58 years). Norm values for QT range between 350 and 400 ms; QTcF values should be <450 ms (men) and <460 ms (women) [25].

Device	QT (ms)			QTcF (ms)		
	Value, mean (SD)	Value, range	Deviation (%), mean (SD)	Value, mean (SD)	Value, range	Deviation (%), mean (SD)
Custo Cardio 300	387.89 (27.17)	325-478	—^b^	399.09 (18.16)	360-445	—
Kardia Mobile	350.62 (26.53)	296-440	9.53 (3.62)	360.05 (22.00)	313-411	9.78 (3.76)
Apple Watch 4	362.00 (27.17)	303-474	6.85 (4.02)	369.57 (22.11)	323-417	7.43 (4.17)

^a^QTcF: QT interval corrected for heart rate using Fridericia formula.

^b^Not available.

### Agreement Between Kardia Mobile and 12-Lead Gold Standard ECG

#### QT interval

**Figure 5:**
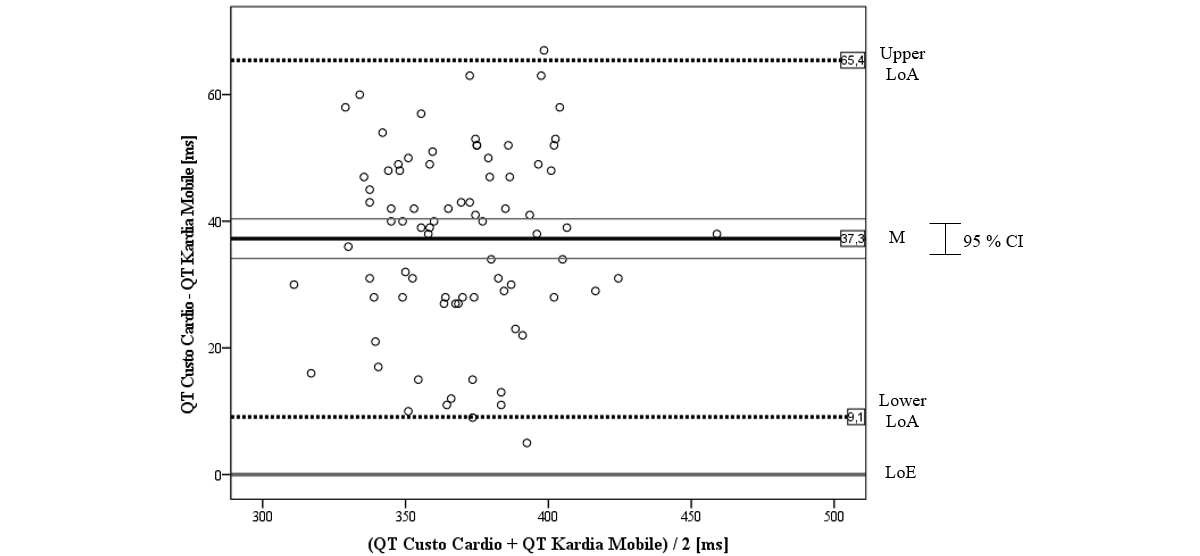
Bland-Altman plot for QT interval of Kardia Mobile versus Custo Cardio. LoA: limit of agreement; LoE: line of equality; M: mean.

For the inferential statistical analysis, we first created a Bland-Altman plot to gain graphical knowledge about the agreement between the Kardia Mobile and Custo Cardio (Figure 5). The mean difference of QT intervals of both ECGs was mean 37.3 (SD 14.4) ms, which is above the LoE. As the LoE is outside the CI of the mean difference, the bias can be considered significant. Furthermore, the LoE lies outside the LoAs, which are SD 28.2 ms around the mean difference. Obviously, there is also 1 outlier above and 1 below the LoAs. In sum, all values are above the LoE; so, it can be concluded that the Kardia ECG tends to systematically underestimate the QT interval compared to the reference device. Finally, as the scatter in the graph is consistent, there seems no discernible trend in relation to the amplitude of the QT interval.

When correlating the measures of both devices, we found a significant positive agreement of *r*=0.857 (P<.001). This result was strengthened by the calculation of *CCC_Lin_*=0.835, which also can be interpreted as almost perfect agreement between the Kardia Mobile and the 12-lead reference.

#### QTcF Interval

**Figure 6:**
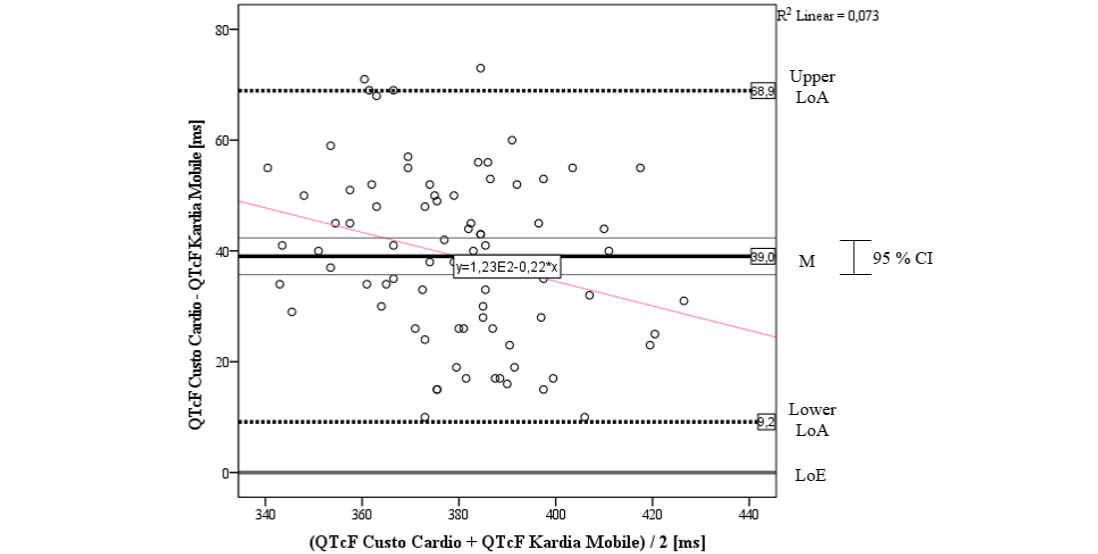
Bland-Altman plot for QTcF interval of Kardia Mobile versus Custo Cardio. LoA: limit of agreement; LoE: line of equality; M: mean; QTcF: QT interval corrected for heart rate using Fridericia formula.

The Bland-Altman plot for the QTcF interval comparing the Kardia Mobile and Custo Cardio graphically showed an obvious deviation, although nearly all values are within the LoAs (Figure 6). The average difference of the QTcF intervals of the ECGs was mean 39.0 (SD 15.3) ms, which is above the LoE. Again, the bias can be considered significant as the LoE is outside the CI of the mean difference. The LoE also lies outside the LoAs, which are SD 34.3 ms around the mean difference. Notably, all values are positive and none comes close to the LoE. The scatter seems to increase in the low and high range of QTcF intervals. In sum, it can be concluded that the Kardia Mobile seems to systematically underestimate QTcF intervals compared to the 12-lead reference. Furthermore, we found a slight trend in dispersion, indicated by the pink regression line, whereby the deviation seems to decrease with increasing QTcF interval.

When correlating the measures of both devices, we found a significant positive agreement of *r*=0.727 (P<.001). This result was strengthened by the calculation of *CCC_Lin_*=0.682, which also can be interpreted as strong agreement between Kardia Mobile and the 12-lead reference.

#### Measurement Error

In addition to the accuracy of the measurements, we tested whether the deviation of the compact ECG increased with increasing heart rate. Therefore, we computed the deviation as a quotient subtracted from 1 and correlated it with the heart rate. For QT intervals, we found a small negative but significant effect of *r*=–0.291 (P<.01), while for QTcF intervals, the effect was even smaller and not significant (*r*=–0.181; P>.05). Thus, the hypothesis that the deviation of the measurement error of the Kardia Mobile might increase with increasing heart rate could be disclaimed for both QT and QTcF intervals.

### Agreement Between Apple Watch and 12-Lead Gold Standard ECG

#### QT Interval

**Figure 7:**
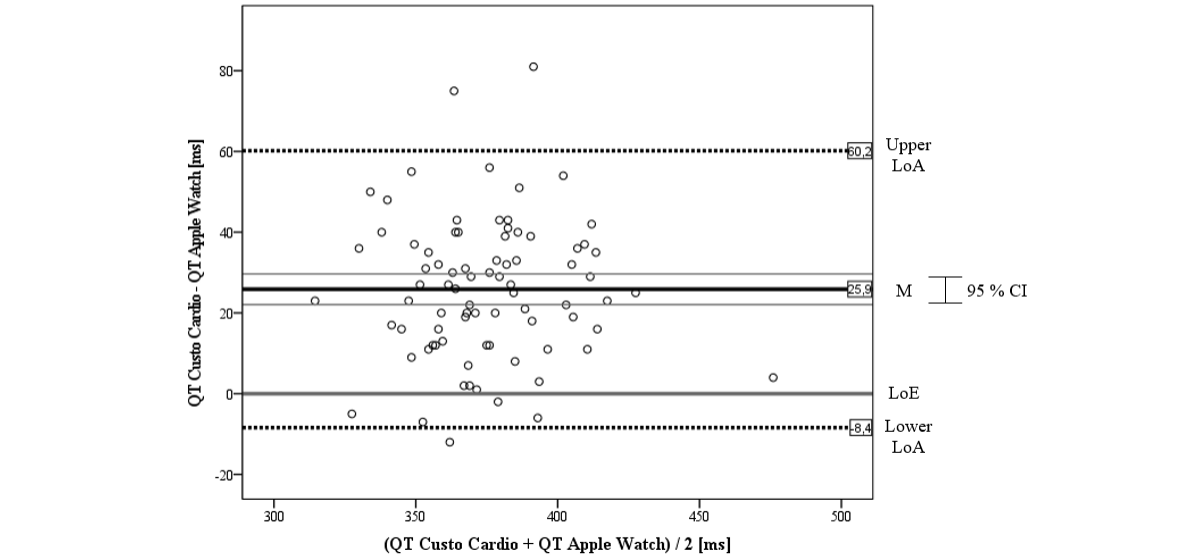
Bland-Altman plot for QT interval of Apple Watch versus Custo Cardio. LoA: limit of agreement; LoE: line of equality; M: mean.

Also, for the Apple Watch, we began the inferential statistics with the graphical analysis via the Bland-Altman plot for QT intervals comparing the Apple Watch with Custo Cardio (Figure 7). On average, the difference of the ECGs was mean 25.9 (SD 17.5) ms, which is above the LoE. Here, the bias is apparently smaller than the one of the Kardia Mobile but can also be considered significant as the LoE is outside the CI of the mean difference. In contrast to the Kardia Mobile, the LoE is within the LoAs, which are SD 34.3 ms around the mean difference. Although the range of the LoAs is the highest, a total of 3 outliers could be identified in the figure. Unlike the Kardia ECG, not all values are above the LoE and 5 values are negative. Another difference is the plotting of some values near the zero line, which indicates their approximate agreement. Nevertheless, in sum, it can be concluded the Apple Watch also tends to systematically underestimate the QT intervals compared to the 12-lead reference. Finally, as the scatter in the graph is consistent, there seems to be no trend in relation to the amplitude of the QT interval.

When correlating the measures of both devices, we found a significant positive agreement of *r*=0.793 (P<.001). This result was strengthened by the calculation of *CCC_Lin_*=0.779, which also can be interpreted as almost perfect agreement between the Apple Watch and the 12-lead reference.

#### QTcF Interval

**Figure 8:**
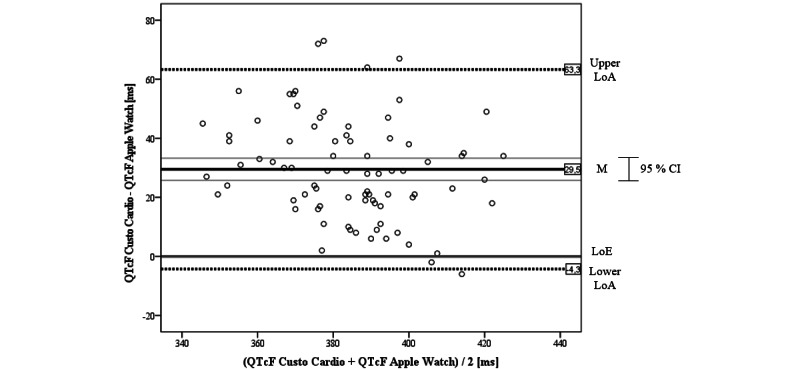
Bland-Altman plot for QTcF interval of Apple Watch versus Custo Cardio. LoA: limit of agreement; LoE: line of equality; M: mean; QTcF: QT interval corrected for heart rate using Fridericia formula.

The Bland-Altman plot for the QTcF interval comparing the Apple Watch and Custo Cardio showed an obvious deviation with some values outlying LoAs (Figure 8). The mean difference of the ECGs was mean 29.5 (SD 17.2) ms, which is above the LoE. As the LoE is outside the CI of the mean difference, the bias can be considered significant. Although the deviation is clearly visible, the LoE is within the LoAs, which are SD 33.8 ms around the mean difference and are similar to the LoAs of the QT interval. There are 4 outliers above the upper LoA and 1 below the lower LoA. Except for 2 values, all values are above the LoE. The few values touching the LoE reached the highest agreement. In sum, it can be concluded that the Apple Watch systematically underestimates the QTcF intervals compared to the 12-lead reference. Again, as the scatter is consistent, there seems to be no trend related to the amplitude of the QTcF interval.

When correlating the measures of both devices, we found a significant positive agreement of *r*=0.649 (P<.001). This result was strengthened by the calculation of *CCC_Lin_*=0.615, which also can be interpreted as a strong agreement between the Apple Watch and the 12-lead reference.

#### Measurement Error

Regarding the measurement error in correlation with increasing heart rate, again, some very small effects could be calculated. For QT intervals, we found a negative significant effect of *r*=–0.289 (P<.01), while for QTcF intervals, the effect was a little smaller but also negatively significant (*r*=–0.231; P<.05). Thus, when relying on the small, but observable, statistical significance, a relationship between the increasing deviation of the measurement error of the Apple Watch by increasing heart rate seems possible.

## Discussion

### Principal Findings

This study aimed to validate mobile single-lead ECGs compared to the 12-lead gold standard. Basically, standard QT measurement uses the longest QT interval of the 12-lead ECG. This is mostly lead II. The single-lead ECG of the Kardia Mobile and the Apple Watch are obtained in a somewhat different position. However, referring to Salvi et al [35], an alternative lead can be used, that is, if lead II is not available. Lead I seems appropriate for determining the QT interval.

According to our findings, the ECG of the Kardia Mobile differed in the measured QT as well as QTcF intervals by an average of less than 10% from the reference device. The deviations in both parameters could be seen clearly in the Bland-Altman plots. Thus, the Kardia Mobile tended to systematically underestimate both parameters. While there was no discernible trend in the magnitude of the difference in the QT interval, the underestimation seemed to decrease slightly with increasing QTcF time. The measured QT values correlated significantly and strongly positive with each other and also showed an almost perfect agreement to the Lin correlation coefficient. The calculated QTcF values generally showed a somewhat lower correlation and a slightly larger deviation compared to the reference device. Further, the correlation was numerically lower but can still be interpreted as a strong agreement, that is, based on our results it can be assumed that the Kardia Mobile measures concordantly when compared to the 12-lead reference device.

However, relating this finding to the current state of research seems problematic as most of the studies examine the general sensitivity (true positive) and specificity (true negative) of the diagnosis of atrial fibrillation but not the measurement accuracy of the different parameters themselves. The sensitivity of the detection of atrial fibrillation ranges from 54.5% to 100%, with most studies calculating values of >87% [36,37]. In 2019, the National Institute for Health and Care Excellence examined in a review comprising 9 studies the accuracy of 4 single-lead ECGs (Kardia Mobile, imPulse, MyDiagnostick, and Zenicor-ECG) [38]. All devices were tested for their sensitivity and specificity of detecting atrial fibrillation. In total, the devices reached a pooled sensitivity of 90.8% (95% CI 83.8%-95%) and a pooled specificity of 95.6% (95% CI 89.4%-98.3%). The validity of Kardia Mobile has been confirmed by 4 studies in this review. It gained a pooled sensitivity of 94% (95% CI 85.1%-97.7%) and a pooled specificity of 96.8% (95% CI 88%-99.2%). The single-lead ECGs also have an algorithm that enables them to deduce diagnostic suggestions. The accuracy of this algorithm was reviewed in the paper of National Institute for Health and Care Excellence [38], too. Here, a sensitivity of 88% (95% CI 32.3%-99.1%) and a specificity of 97.2% (95% CI 95.1%-98.5%) was found for the Kardia Mobile. However, future studies are needed, as the evidence of the available studies is not sufficient for recommending the routine adoption of single-lead ECG devices for atrial fibrillation detection [38]. According to Hnatkova et al [39], the differences that are still tolerable for the clinical context are SD 15 ms. In our study, the Kardia Mobile excelled this cutoff by more than twice. However, in sports science or in general in nonclinical conditions, setting such high standards as in the clinical context is obsolete. Suitable reference values, that is, to what extent deviations are still within the tolerance range and from when the measurement difference is too high to obtain usable results, should be developed. Another aspect that affects the usability of such devices is their manageability. We experienced problems in approximately 20% (16/81) of the measurements with the Kardia Mobile. Often the measurements were interrupted due to a lack of connection, so several trials were necessary wherein the finger position or the contact pressure was varied. Furthermore, the Kardia Mobile has no display of its own and can only be used together with a smartphone, which in turn needs to be close to the ECG device. Contrary to the manufacturer’s claimed 30-cm distance, according to our own experiences, a distance of 10 cm to the smartphone should not be exceeded. Especially in a lying position, it is problematic to find an optimal positioning of the ECG in which no artifacts due to little finger muscle contractions occur. We suggest sitting at a table for the measurement, but we were unable to do so for the sake of a standardized comparison of measurement methods in this study. When looking at the Apple Watch, we also found a difference in both parameters from the reference device. Compared to the Kardia Mobile, this corresponds to a smaller deviation of approximately 28% for QT and 24% for QTcF intervals. However, the ECG of the Apple Watch tends to underestimate the assessed parameters, too. Again, compared to the Kardia Mobile, the Bland-Altman plots showed an about 10 ms lower but still considerable difference. These differences were also significantly higher than the clinically tolerable deviation of 15 ms. Unlike the Kardia Mobile, some of the differences were negative values, and also, some of them could be plotted near the zero line, which in turn refers to a higher agreement. In both cases, no trend in the scatter in terms of a possible time dependency could be found. Regarding the correlation with the reference device, a strong significant effect was computed. In line with the Lin concordance coefficient, the Apple Watch’s ECG agreed almost perfectly with Custo Cardio’s ECG, although correlations were numerically smaller than those of the Kardia Mobile.

Discussing these findings from the Apple Watch with respect to the current state of the literature is even more difficult than from the Kardia Mobile, as barely any representative studies about the smartwatch’s validity exist. The only representative study was sponsored by Apple [40]. Accordingly, the sensitivity of 98.3% and specificity of 99.6% postulated by Apple should be seen critically, since these calculations exclude all ambiguous and nonclassifiable values. If these values were included, the sensitivity would be 90.5% and the specificity 85.2%, which differs significantly from the values primarily mentioned. Regarding Apple’s ECG algorithm, a sensitivity of 95.5% and a specificity of 97.1% was calculated [40]. Thus, also here, suitable reference values for classifying the range of deviation and their tolerance limits are missing and should be considered in future studies. The German Cardiac Society [41] has described the Apple Watch’s ECG function as “a valuable monitoring tool for establishing important information for patients and their doctors.” However, they have also pointed out that results should be attested by experienced health care providers. As single-lead ECGs are somewhat concordant but do not become 100% close to the 12-lead medical standard, those recordings are not able to replace the visit at the doctor’s office, especially in patients with preexisting cardiovascular illness [41].

Furthermore, the aspect of manageability should also be addressed at this point. In about 5% (4/81) of the trials, we had a few problems when recording an ECG with the Apple Watch, so the measurements failed. In significantly more cases (12/81, 15%), difficulties occurred when lying down to find an adequate arm position in which the ECG is not triggered by action potentials of muscle contractions. Again, it is advisable to carry out the measurement at a table in a seated position in order to provide support for the arms. Contrary to the Kardia Mobile, the Apple Watch can record the ECG without a smartphone connection. The visual representation on the watch is simple and easy to understand.

The question of whether the measurement error of the compact ECGs increases with increasing heart rate cannot be denied in total. For all parameters, both for the Kardia Mobile and for the Apple Watch, we found negative correlations with a weak effect. Except for the QTcF time of the Kardia Mobile, the results were also significant. However, it should be kept in mind that most of the participants were young and athletic, which can have an impact on their heart rate.

Overall, compact single-lead ECG devices, for example, for the smartphone or the smartwatch, seem to be a good alternative to the previous standard 12-lead ECGs in terms of algorithmic detection of atrial fibrillation and imaging of the ECG wave [42,43]. Furthermore, the advantage is that the ECG recording need not take place in the doctor’s office. Further, it can easily be sent as a PDF file via an email requesting for evaluation by a specialist personnel. While AliveCor has already conducted few studies regarding the validity of their ECG products, less is known about the Apple Watch’s validity for ECG recording. For example, on the website of AliveCor, a clinical research section with various peer-reviewed papers is available [44]. The problem with many of these studies, however, is that they refer to an “AliveCor device,” which is often not specified in more detail. AliveCor has several ECG-enabled devices on the market such as the Kardia Mobile, the Kardia Mobile 6L, or the Kardia Band for the Apple Watch. Thus, it is unclear in which study which of the devices were proven for their accuracy and usability. As described above, the only validation study of the Apple Watch’s ECG was sponsored and commissioned by Apple [40]. To our knowledge, another representative validation of the device in its ECG function is not available. Thus, further empirical research is needed.

Given the fact that the available consumer technology is proceeding rapidly, the number of smartphone apps and gadgets for recording, visualizing, and evaluating physical performance as well as health data is constantly growing. The greatest effort of such smart devices as the ones used in this study is that they are innovative, reliable, and time- and cost-efficient. Although the mobile compact ECGs seem not to fulfill the validity criteria as medical or clinical diagnostic device, they have a high practical usage potential. The most beneficial or practical use of this new health technology is to be found in home-based health care, especially in terms of cardiovascular disease prevention and health monitoring in everyday life. This is in line with the findings of the increasing number of studies examining the effects of mobile health interventions [45,46]. Finally, some could assume that the role of artificial intelligence systems in health technology (such as the QTc Tracker app used here) will also be greatly increased in the future [47].

### Limitations

There are some constraints limiting our study. As only healthy and mainly young adults took part in this study, the results might be to some extent limited and not generalizable. Further, we assessed the individuals’ ECGs comprehensively but did not include parameters such as the P- or T-wave in our analysis. Therefore, future studies should investigate these parameters as well as the measurement error in dependence on the pulse rate. Further, clustering participants according to a pulse range or to age groups would be interesting. We validated the mobile ECGs in a lying, resting position. Regarding their practical usability, comparable measurements during or after exercising are required. Finally, from a methodological perspective, it should be mentioned that the observed correlations and their statistical significance are limited. Although high correlations are positive findings, they do not necessarily indicate high test accuracy. In line with previous research, we relied on Cohen classification, but his suggestions on acceptable correlations and effect sizes were based on his research in the social sciences, that is, when assessing physiological functions or bioelectrical signals where the value of correlations is discussable. Furthermore, there is a potential bias from multiple comparisons. The most useful and informative data to rely on when determining acceptability of the testing mechanisms rather seem to relate to the Bland-Altman plots. Thus, with regard to a comprehensive methodological and analytical approach as well as in order to strengthen the measurements’ concordance, the combination of correlative or regressive with Bland-Altman analyses is recommended.

### Conclusions

In medicine and science, 12-lead ECGs are the gold standard for cardiovascular diagnostics. As their usability is quite extensive and, beyond that, the technological progress offers smart time- and cost-efficient tools, consumers prefer mobile ECGs in nonclinical conditions. In this study, we thus validated the single-lead ECGs of the Kardia Mobile and the Apple Watch 4. Besides single-digit deviation from the 12-lead reference, concordant ECGs were recorded. To conclude, mobile compact ECGs are an innovative and reliable approach, especially in terms of cardiovascular disease prevention and health monitoring in everyday life. However, to date, they seem not to fulfill the validity criteria as a medical or clinical diagnostic device.

## Abbreviations

bpm: beats per minute
CCCLin: Lin concordance correlation coefficient
ECG: electrocardiogram
FDA: Food and Drug Administration
LoA: limit of agreement
LoE: line of equality
r: Pearson correlation coefficient
QTc: QT interval corrected for heart rate
QTcF: QT interval corrected for heart rate using Fridericia formula

## Data Availability

The data sets generated and analyzed during this study are available from the corresponding author on reasonable request.
